# MYCN upregulates the transsulfuration pathway to suppress the ferroptotic vulnerability in *MYCN*-amplified neuroblastoma

**DOI:** 10.15698/cst2022.02.264

**Published:** 2022-01-17

**Authors:** Konstantinos V. Floros, Ayesha T. Chawla, Mia O. Johnson-Berro, Rishabh Khatri, Angeliki M. Stamatouli, Sosipatros A. Boikos, Mikhail G. Dozmorov, L. Ashley Cowart, Anthony C. Faber

**Affiliations:** 1 Department of Oral and Craniofacial Molecular Biology, Philips Institute for Oral Health Research, VCU School of Dentistry and Massey Cancer Center, Virginia Commonwealth University, Richmond, VA, USA.; 2 Division of Endocrinology, Diabetes, and Metabolism, Department of Internal Medicine, Virginia Commonwealth University School of Medicine, Richmond, VA, USA.; 3 Division of Hematology, Oncology and Palliative Care, Virginia Commonwealth University and Massey Cancer Center, Richmond, VA, USA.; 4 Department of Biostatistics, Virginia Commonwealth University, Richmond, VA, USA.; 5 Department of Biochemistry and Molecular Biology and Massey Cancer Center, Virginia Commonwealth University, Richmond, VA, USA.; 6 Hunter Holmes McGuire Veteran’s Affairs Medical Center, Richmond, VA, USA.

**Keywords:** MYCN-amplified neuroblastoma, ferroptosis, cystathionine, transsulfuration pathway, methionine, cysteine

## Abstract

Ferroptosis is an iron-dependent, oxidative form of cell death that is countered mainly by glutathione peroxidase 4 (GPX4) and the production of glutathione (GSH), which is formed from cysteine. The identification of the cancers that may benefit from pharmacological ferroptotic induction is just emerging. We recently demonstrated that inducing ferroptosis genetically or pharmacologically in *MYCN*-amplified neuroblastoma (NB) is a novel and effective way to kill these cells. MYCN increases iron metabolism and subsequent hydroxyl radicals through increased expression of the transferrin receptor 1 (TfR1) and low levels of the ferroportin receptor. To counter increased hydroxyl radicals, MYCN binds to the promoter of *SLC3A2* (solute carrier family 3 member 2). SLC3A2 is a subunit of system Xc-, which is the cysteine-glutamate antiporter that exports glutamate and imports cystine. Cystine is converted to cysteine intracellularly. Here, we investigated other ways MYCN may increase cysteine levels. By performing metabolomics in a syngeneic NB cell line either expressing MYCN or GFP, we demonstrate that the transsulfuration pathway is activated by MYCN. Furthermore, we demonstrate that *MYCN*-amplified NB cell lines and tumors have higher levels of cystathionine beta-synthase (CBS), the rate-limiting enzyme in transsulfuration, which leads to higher levels of the thioether cystathionine (*R-S*-(2-amino-2-carboxyethyl)-l-homocysteine). In addition, *MYCN*-amplified NB tumors have high levels of methylthioadenosine phosphorylase (MTAP), an enzyme that helps salvage methionine following polyamine metabolism. MYCN directly binds to the promoter of *MTAP*. We propose that MYCN orchestrates both enhanced cystine uptake and enhanced activity of the transsulfuration pathway to counteract increased reactive oxygen species (ROS) from iron-induced Fenton reactions, ultimately contributing to a ferroptosis vulnerability in *MYCN*-amplified neuroblastoma.

## INTRODUCTION

Both the evasion of cell death and dysregulated metabolism are hallmarks of cancer [1]. Apoptosis has been the most studied type of cell death that tumors circumvent, and apoptotic-inducing BH3 mimetics have revolutionized hematological cancer therapy [2, 3]. More recently, a form of non-apoptotic cell death with important metabolic cross-talks, termed ferroptosis, has been described [4]. Ferroptosis is characterized by the iron (Fe)-dependent accumulation of reactive oxygen species (ROS), leading to excessive lipid peroxidation and cell death [4, 5].

Rate-limiting accumulation of iron is at the receptor levels - the transferrin receptor protein 1 (TfR1) is responsible for iron import, while ferroportin (FPN) is responsible for iron export [6]. We discovered that *MYCN*-amplified neuroblastoma (NB) tumors have high levels of TfR1 and low levels of FPN, contributing to increased cellular iron levels [7]. Iron contributes to a number of important biological pathways that promote growth/survival of cancer cells, making it perhaps not surprising that iron consumption (for instance, in red meat) correlates with cancer risk [8]. Importantly, in addition to its contributions to biological pathways, iron also increases cellular ROS through the Fenton reaction, leading to the generation of lipid peroxides. The accumulation of these lipid peroxides is counteracted by the glutathione (GSH)-dependent peroxidase, GPX4, which converts lipid peroxides into non-toxic lipid alcohols [9]. GSH synthesis requires cysteine. Cystine is the major extracellular resource of what eventually becomes cysteine. The cell controls extracellular cystine intake through an antiporter receptor, where cystine enters as glutamate exits. This cystine-glutamate antiporter is composed of a light chain subunit, the 12-pass transmembrane protein, solute carrier family 7 member 11 (SLC7A11) (Xc-), and a heavy chain subunit, the single-pass transmembrane protein solute carrier family 3 member 2 (SLC3A2). Cysteine can also be produced independent of this pathway *de novo* through the transsulfuration pathway [10]. We recently reported that MYCN sensitizes NB to cystine withdrawal and MYCN binds to and upregulates *SLC3A2* and increases GSH levels [7], presumably to increase cysteine to counteract increased ROS from Fenton reactions. Here, we perform mass spectrometry metabolomics to better understand the regulation of cysteine by MYCN in NB, and to determine whether the transsulfuration pathway contributes cysteine.

## RESULTS

**Figure 1:**
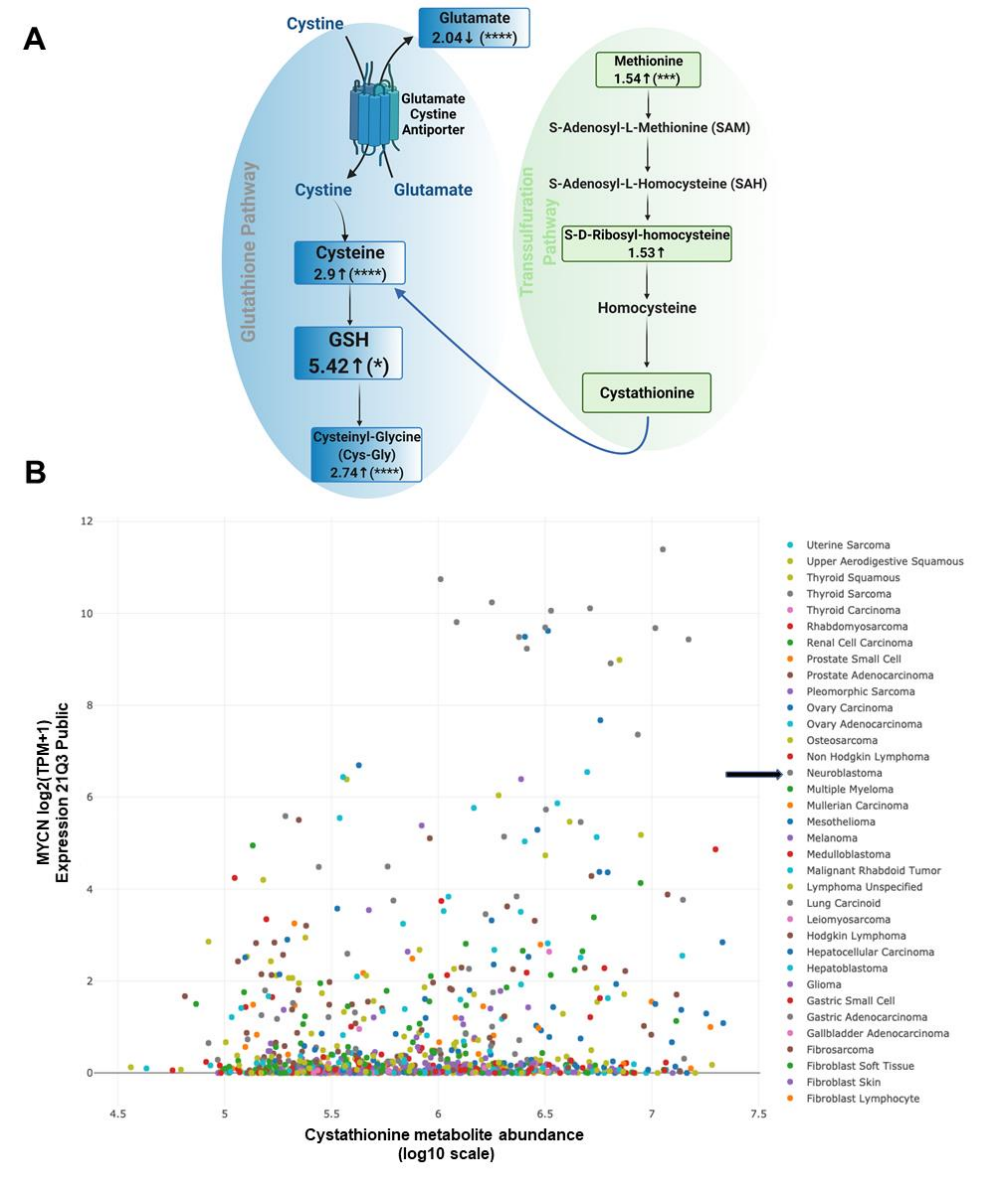
FIGURE 1: Overexpression of MYCN leads to high levels of cystathionine. **(A)** Changes in key metabolites of the glutathione and the transsulfuration pathway after analyzing SK-N-SH MYCN and GFP cells by untargeted liquid chromatography/tandem mass spectrometry. Samples were run in quintuplicate. For high throughput/untargeted metabolomics, compounds are resolved by liquid chromatography and then analyzed for mass and fragmentation by tandem mass spectrometry. In complex biological mixtures such as cell lysates, the liquid chromatography-based resolution is not optimized for any one metabolite, and therefore some metabolites of interest (e.g., cystathionine) may not be readily identified. The numbers represent the log_2_ (fold change) of the metabolites in SK-N-SH MYCN compared to SK-N-SH GFP cells; p-values were determined by t-test with false discovery rate. **(B)** DEPMAP (Broad Institute consortium) analysis of the correlation between cystathionine and *MYCN* expression among 910 cancer cell lines of all cancer subtypes. For all statistical considerations differences were considered statistically significant if P < 0.05. For all calculated P values: ^*^, P < 0.05; ^**^, P < 0.01; ^***^, P < 0.001; ^****^, P < 0.0001.

We employed our syngeneic SK-N-SH neuroblastoma cell pair expressing exogenous MYCN or GFP [11] and performed untargeted liquid chromatography/tandem mass spectrometry of cell lysates to determine changes in a broad array of polar metabolites. Our data indicated decreased glutamate levels, increased cysteine levels and increased cysteinyl-glycine (Cys-Gly) levels, consistent with an activated system Xc-antiporter [7]. ’Confirming our previously published data from colorimetric analyses of GSH levels [7], GSH levels were increased over 5-fold in the presence of MYCN (**Fig. 1A**).

**Figure 2:**
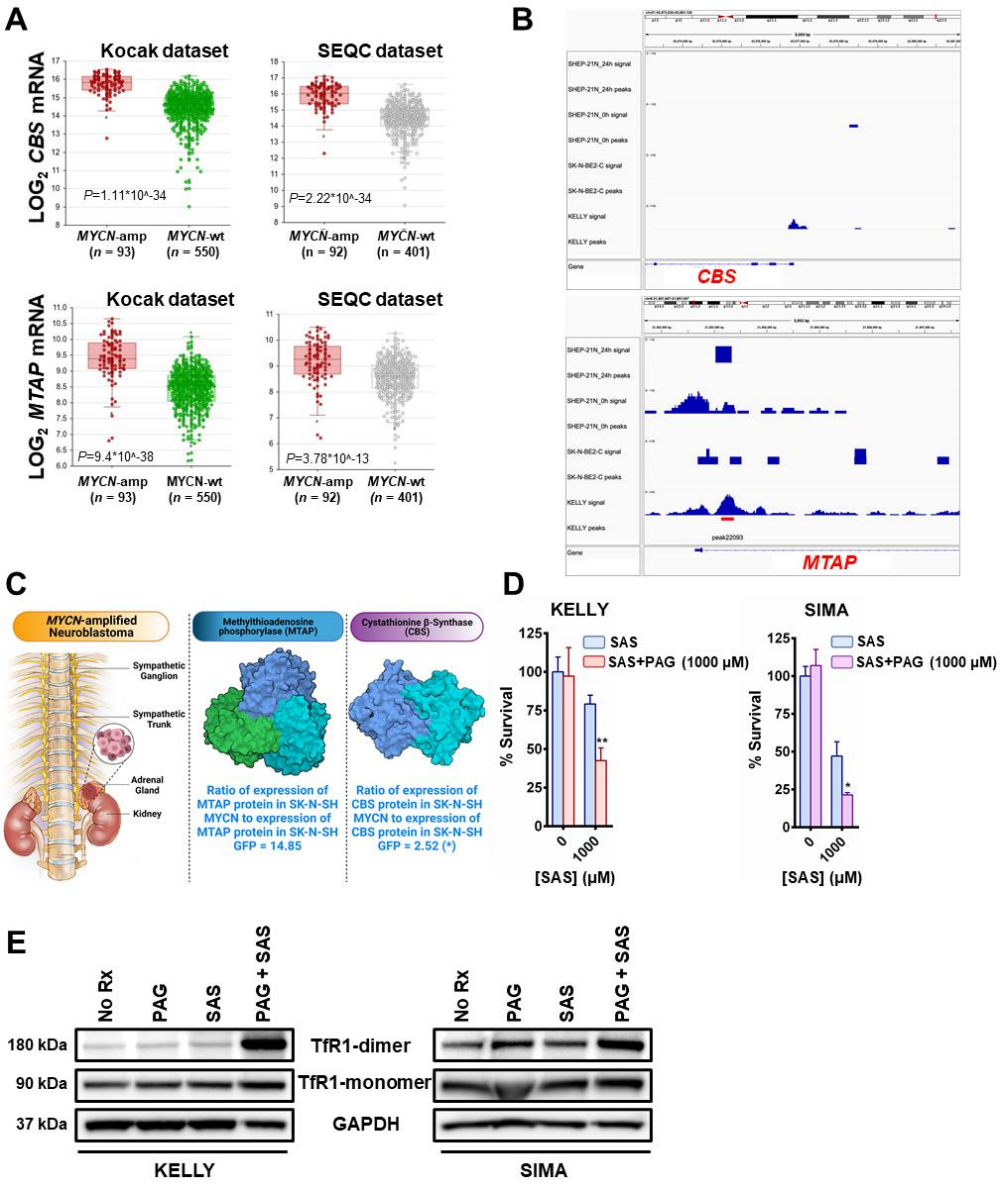
FIGURE 2: MYCN induces the transsulfuration pathway to further protect neuroblastomas from ferroptotic cell death. **(A)** Box plots from datasets obtained from R2 platform demonstrating differential RNA expression of *CBS* and *MTAP* enzymes in *MYCN*-amplified neuroblastoma tumors compared with *MYCN*-wt neuroblastoma tumors. Mann–Whitney test was performed. **(B)** MYCN signal in the promoters of *MTAP* and *CBS* genes in the corresponding neuroblastoma cell lines. 24 h - signal post MYCN shutdown. Signal tracks were scaled to the same range for comparison. Red bar represents statistically significant MYCN binding in the *MTAP* promoter and lack of binding in the *CBS* promoter. **(C)** Fold-change in the expression levels of CBS and MTAP proteins between SK-N-SH MYCN and SK-N-SH GFP cells analyzed by LC-MS. Three biological replicates have been used for the mass spec analysis. For statistical significance ANOXA test was performed. The data has been included in supplemental material (Table 1S). For the 3D structures of MTAP and CBS molecules the Protein Data Bank (PDB) was used. More specifically, for the MTAP molecule, PDB ID: 3OZE [36] and for the CBS molecule, PDB ID: 1JBQ [37]. **(D)** The *MYCN*-amplified neuroblastoma cell lines KELLY and SIMA were treated with 0 and 1000 μM of sulfasalazine (SAS) with or without 1mM propargylglycine (PAG) for 36 h (KELLY) and 24 h (SIMA) and cell viability was assessed by CellTiter-Glo (*n*=3; error bars, +SD). **(E)** Whole-cell lysates were prepared from KELLY and SIMA cells that were treated overnight with (i) no drug (No Rx), (ii) 1mM propargylglycine (PAG), (iii) 1mM sulfasalazine (SAS) and (iv) their combination, subjected to western blotting, and probed for the indicated proteins. For **D**, Student *t* test was performed, and P values were corrected for multiple testing using Bonferroni method. For all statistical considerations differences were considered statistically significant if P < 0.05. For all calculated P values: *, P < 0.05; **, P < 0.01.

Importantly, there was also evidence of increased transsulfuration pathway activation by MYCN. The transsulfuration pathway transfers a sulfur group from methionine for cysteine biosynthesis. Methionine is an essential sulfur containing amino acid that is acquired from the diet and converted to the ubiquitous methyl donor, S-adenosyl-methionine (SAM). SAM donates its methyl group and is thus converted to S-adenosyl-homocysteine (SAH). SAH hydrolysis results in the production of homocysteine, which can then be converted to cystathionine and then to cysteine via the transsulfuration pathway (**Fig. 1A**). Of note, homocysteine can also pick up a methyl group from the folate cycle, and return as methionine [12]. Cystathionine is formed as an intermediate during transsulfuration via the condensation of homocysteine by cystathionine β-synthase (CBS). While we did not detect cystathionine by mass spectrometry in these samples, we evaluated the DepMap portal metabolites dataset where cystathionine was quantified across hundreds of cancer cell lines. Consistent with a role of MYCN increasing the transsulfuration pathway, cancer cell lines with high MYCN demonstrated high levels of cystathionine (*r* = 0.199), which likely is MYCN driven as other high MYCN cancer cell lines of different tissue origins (e.g., small cell lung cancer) had high levels as well (**Fig. 1B**). This was consistent with a substantial increase of CBS in *MYCN*-amplified NB compared to wild-type tumors (**Fig. 2A**), however was not the result of MYCN binding to the promoter of CBS (**Fig. 2B**).

**Figure 3:**
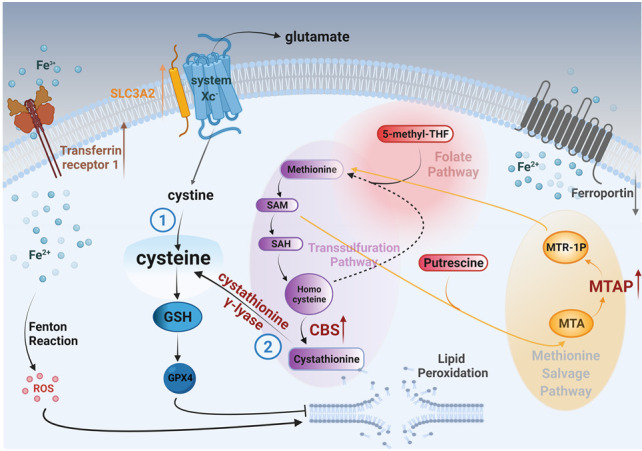
FIGURE 3: Suggested model for the metabolic pathways that lead to accumulation of cysteine and ultimately to glutathione (GSH) and inhibition of lipid peroxidation in *MYCN*-amplified neuroblastomas. Cysteine can derive either (1) from cystine that is imported by system xc-, or (2) from cystathionine that is produced from methionine through the transsulfuration pathway. To provide adequate fuel molecules to the transsulfuration pathway, MYCN orchestrates the induction of the key enzymes CBS and MTAP.

Methionine can also be produced through a methionine salvage pathway generated from polyamine metabolism. Here, methylthioadenosine (MTA) is converted by the enzyme MTA phosphorylase (MTAP) to 5-methylthioribose-1-phosphate (MTR-1P), a precursor of methionine. We found MTAP was highly elevated in *MYCN*-amplified NB tumors [13, 14] (**Fig. 2A**) and, confirmed an earlier report that MYCN binds the promoter of MTAP [15] (**Fig. 2B**). These data demonstrate MYCN binds to the promoter of MTAP to increase salvage of methionine, likely contributing to sustained methionine levels which could perpetuate cysteine biosynthesis. Mass spectrometry in the same syngeneic SK-N-SH cell line confirmed increased expression of CBS (2.52-fold) and MTAP (14.85-fold) (**Fig. 2C** and Table S1). Treatment with propargylglycine (PAG) [16], a pharmacological inhibitor of the key enzyme in the transsulfuration pathway that catalyzes the hydrolysis of cystathionine into cysteine, cystathionine γ-lyase (CSE), was sufficient to sensitize two *MYCN*-amplified NB cell lines to system Xc-inhibition (**Fig. 2D**). Significant accumulation of the dimeric form of TfR1 [17] in the presence of both inhibitors evidenced ferroptosis (**Fig. 2E**) [18]. Our results from this study and our previous one [7] are summed up in **Fig. 3**.

## DISCUSSION

To suppress oxidative lipid damage and ferroptosis, our data reveal that *MYCN*-amplified NB employs an overactive GSH pathway, which is at least in part due to a direct result of MYCN upregulation of SLC3A2 [7] important for stabilization of system Xc- [19].

Here we performed select metabolic profiling in an NB cell line that was engineered to expresses exogenous MYCN or GFP, to determine other sources of cysteine in the cell that could help counteract ROS. We found higher levels of metabolites involved in the transsulfuration pathway and confirmed in cell lines there are high levels of cystathionine across different tissues of high MYCN-expressing cancers. Consistent with an oncogene effect, we found strikingly high levels of CBS, the enzyme that forms cystathionine and the rate-limiting enzyme in transsulfuration in the *MYCN*-amplified NB tumors. Interestingly, CBS catalyzes the condensation of serine and homocysteine (Hcy) to form cystathionine. Serine synthesis has been reported to be important for *MYCN*-amplified NB, once again a result of direct MYCN upregulation of the key enzymes in the pathway [20]. We also observed high MTAP expression, an enzyme in the methionine salvage pathway that converts the polyamine nucleoside byproduct MTA, to a methionine precursor. Polyamine catabolism is an important growth/survival pathway in NB [21]. In fact, ornithine decarboxylase carnitine (ODC) is upregulated by MYCN, leading to upregulation of polyamine biosynthesis [22]. In addition, while *MYCN*-amplified NB tumors have increased expression of both system Xc-components compared to MYCN wild-type tumors (SLC7A11 and SLC3A2); however, both are relatively lowly expressed in NB compared to other tumors (ref. [23] and the DepMap portal, depmap.org), which suggested to us that there may be other contributions to cysteine biosynthesis. Indeed, treatment with a pharmacological inhibitor of PAG with sulfasalazine (SAS) led to enhanced ferroptosis (**Fig. 2E**) evidencing an additive role of the transsulfuration pathway to help detoxify ROS from iron metabolism in *MYCN*-amplified NB.

Noteworthy, the cell lines used in the current study were maintained in regular CO_2_ incubators with an oxygen tension matching that of atmospheric oxygen levels (∼20%). This is much higher than the oxygen levels experienced *in vivo* (∼5%). Exposure to more physiological oxygen conditions might partially affect the data collected from our metabolomic experiments [24].

How may these results be clinically translated? Buthionine sulfoximine (BSO), an inhibitor of γ-glutamylcysteine synthetase (γ-GCS), the rate limiting enzyme in GSH synthesis, effectively reduces GSH in NB cell lines [7, 25]. BSO was investigated recently in a phase I trial in combination with melphalan. BSO was administered as a 3 g/m^2^ bolus and was well tolerated in 28 patients with recurrent/refractory NB [26]. As such, BSO is likely currently the most compelling drug to translate in a biomarker-directed (i.e., *MYCN* amplification) way. How do we target the transsulfuration pathway in combination with BSO? One such interesting possibility is the experimental recombinant methioninase (rMETase) which blocks methionine dependent xenografts and have been dosed successfully in patients [27].

In conclusion, MYCN increases cystathionine production and directly upregulates MTAP, shunting methionine into the transsulfuration pathway, contributing cysteine. Thus, in addition to system Xc-receptor activation, this pathway provides another source for cysteine metabolism in *MYCN*-amplified NB. Targeting this part of the pathway in conjunction with canonical inhibitors like BSO may be an exciting avenue of therapy for *MYCN*-amplified NB, and advances our understanding of how MYCN meets the enhanced GSH reliance in *MYCN*-amplified neuroblastoma.

## MATERIALS AND METHODS

### Cell lines

The cell lines SK-N-SH, SIMA and KELLY were from the Molecular Center Therapeutics Laboratory at Massachusetts General Hospital (Boston, MA), which performs routine testing of cell lines by single-nucleotide polymorphism and short tandem repeat analysis. The SK-N-SH cell line was cultured in DMEM/F12 (50:50) supplemented with 10% FBS, 1 μg/mL penicillin and streptomycin. The SIMA and KELLY cell lines were cultured in RPMI 1640 supplemented with 10% FBS, 1 μg/mL penicillin and streptomycin. All three cell lines were used for less than 40 passages but were not independently authenticated. They were regularly screened for *Mycoplasma* using a MycoAlert Mycoplasma Detection Kit (LT07–318; Lonza). All cell lines were maintained in a CO_2_ incubator under regular cultured conditions (37^o^C, 5% CO_2_, ∼20% oxygen tension)

### Reagents and antibodies

The following reagents were purchased: Sulfasalazine (SAS) (S0883; Sigma-Aldrich), DL-Propargylglycine (PAG) (P7888; Millipore Sigma). The antibodies used for immunoblotting in this study were as follows: anti-GAPDH (sc-32233; Santa Cruz), Transferrin Receptor Monoclonal Antibody (H68.4) (13-6800; ThermoFisher Scientific).

### Western blotting

Cells were lysed in lysis buffer (20 mM Tris, 150 mM NaCl, 1% Nonidet P-40, 1 mM EDTA, 1 mM EGTA, 10% glycerol, and protease and phosphatase inhibitors), incubated on ice for 15 min, and centrifuged at 16,000 × g for 10 min at 4 °C. Equal amounts of the detergent-soluble lysates were resolved using the NuPAGE Novex Midi Gel system on 4–12% Bis–Tris gels (Invitrogen), transferred to PVDF membranes (PerkinElmer) in between six pieces of Whatman paper (Fisher Scientific) set in transfer buffer from Biorad with 20% methanol, and following transfer and blocking in 5% nonfat milk in PBS, probed overnight with the antibodies listed above. Representative blots from several experiments are shown in the figures. Chemiluminescence was detected with the Syngene G: Box camera (Synoptics).

### Cell viability assay

The CellTiter-Glo experiments were performed in 96-well flat-bottom black plates. Cells were treated with 25 μL of CellTiter-Glo (G7573; Promega), following continuous drug treatment (each time with the indicated drugs at the indicated concentrations), at 37°C and 5% atmospheric CO_2_ and immediately read on a H1 Biotek plate reader according to the Promega protocol. Quantification of non-treatment seeded cells was used to determine the total cell growth number over the experiment.

### Vector construction and establishing stable cell lines

The pLENTI-GFP control plasmid was previously described [28]. The plasmid pMXs-hu-*N-Myc* [29] was a gift from Shinya Yamanaka (50772; Addgene) and cloned into the pLENTI backbone to form pLENTI-*MYCN*. SK-N-SH cells were transduced with plasmid-containing viral particles and viral particles were generated in 293T cells and collected after 48 h.

### Database analyses

Gene expression datasets were obtained from the R2:Genomics analysis and visualization platform (http://r2.amc.nl), which contains data from the tumor neuroblastoma datasets including: Kocak [30] and SEQC [31]. These datasets contain mRNA expression data. The analysis was performed with R2, and the data and p-values were downloaded. MYCN/Cystathionine correlation data were obtained from DepMap consortium (https://depmap.org/portal/). For Figure 1D: MYCN ChIP-seq hg38 data were obtained from Cistrome DB [32]. Data for SHEP21 and KELLY neuroblastoma cell lines (Cistrome DB: 84181, 84176, 88303) were from Zeid et al. study [33]. Data for SK-N-BE(2)-C neuroblastoma cell line (Cistrome DB: 69364) were from Hsu et al. study [34]. IGV browser was used to visualize ChIP-seq signal [35].

### LC-MS analysis

The samples (SK-N-SH MYCN/GFP) were solubilized using a Bead Beater, then reduced with 10 mM DTT in 0.1 M ammonium bicarbonate and then alkylated with 50 mM iodoacetamide in 0.1 M ammonium bicarbonate (both room temperature for 0.5 h). The samples were then digested overnight at 37°C with 0.5 μg trypsin in 50 mM ammonium bicarbonate. The samples were acidified with acetic acid to stop digestion and then spun down and further purified using C18 tips. The solutions were evaporated to 20 μL for MS analysis.

The LC-MS system consisted of a Thermo Exploris 480 mass spectrometer system with an Easy Spray ion source connected to a Thermo 75 μm × 15 cm C18 Easy Spray column. 5 μL of the extracts were injected and the peptides eluted from the column by an acetonitrile/0.1 M formic acid gradient at a flow rate of 0.3 μL/min over 2.0 hours. The nanospray ion source was operated at 1.9 kV. The digest was analyzed using the rapid switching capability of the instrument acquiring a full scan mass spectrum to determine peptide molecular weights followed by product ion spectra (Top10 HCD) to determine amino acid sequence in sequential scans. This mode of analysis produces approximately 25000 MS/MS spectra of ions ranging in abundance over several orders of magnitude. Not all MS/MS spectra are derived from peptides. The data were analyzed by database searching using the Sequest search algorithm against Uniprot Human. The samples (SK-N-SH MYCN/GFP) produced identifications for ∼4000 proteins. The samples were grouped in triplicate by condition and ANOVA analysis was performed.

### Metabolomics

MYCN or GFP-expressing SK-N-SH cells were snap-frozen. Pellets were resuspended in 20% methanol/water and cells were lysed via sonication. Samples were centrifuged for 5 minutes at 5,000 × g to pellet insoluble material. Supernatant was analyzed by liquid chromatography/tandem mass spectrometry using the ThermoFisher Q-Exactive HF system. Cell lysates were resolved by liquid chromatography on a Vanquish UHPLC system on a silica column using a gradient from 50:50 acetonitrile:water with 0.1% formic acid to 1:99 acetonitrile: water with 0.1% formic acid at a flow rate of 300 μl/min, or on a HILIC column using a 50:50 acetonitrile:water with 0.1% formic acid and 5mM ammonium formate 50:50 methanol:water with 0.1% etc.

Samples were analyzed in both positive and negative ion mode. In each ion mode, aliquots of each sample were pooled to generate representative MS2 spectra. Compound Discoverer v. 3.1. was used to deconvolute raw LC/MS data with respect to alignment and peak area determination. Compounds were identified relative to the MS2 spectra from the pooled sample. Statistical analysis was performed by Compound Discoverer v. 3.1. Samples were run in quintuplicate. For high throughput/untargeted metabolomics, compounds are resolved by liquid chromatography and then analyzed for mass and fragmentation by tandem mass spectrometry. In complex biological mixtures such as cell lysates, the liquid chromatography-based resolution is not optimized for any one metabolite, and therefore some metabolites of interest (e.g., cystathionine) may not be readily identified.

## SUPPLEMENTAL MATERIAL

Click here for supplemental data file.

All supplemental data for this article are available online at https://www.cell-stress.com/researcharticles/2022a-floros-cell-stress/.

## Abbreviations:

BSO: – buthionine sulfoximine,
CBS: – cystathionine-beta-synthase,
FPN: – ferroportin,
GSH: – glutathione,
MTA: – methylthioadenosine,
MTAP: – MTA phosphorylase,
NB: – neuroblastoma,
PAG: – propargylglycine,
ROS: – reactive oxygen species,
SAH: – S-adenosyl-homocysteine,
SAM: – S-adenosyl-methionine.

## References

[1] Hanahan D, Weinberg RA (2011). Hallmarks of cancer: the next generation.. Cell.

[2] Souers AJ, Leverson JD, Boghaert ER, Ackler SL, Catron ND, Chen J, Dayton BD, Ding H, Enschede SH, Fairbrother WJ, Huang DC, Hymowitz SG, Jin S, Khaw SL, Kovar PJ, Lam LT, Lee J, Maecker HL, Marsh KC, Mason KD, Mitten MJ, Nimmer PM, Oleksijew A, Park CH, Park CM, Phillips DC, Roberts AW, Sampath D, Seymour JF, Smith ML (2013). ABT-199, a potent and selective BCL-2 inhibitor, achieves antitumor activity while sparing platelets.. Nat Med.

[3] Rossi D (2016). Venetoclax: a new weapon to treat high-risk CLL.. Lancet Oncol.

[4] Dixon SJ, Lemberg KM, Lamprecht MR, Skouta R, Zaitsev EM, Gleason CE, Patel DN, Bauer AJ, Cantley AM, Yang WS, Morrison B, Stockwell BR (2012). Ferroptosis: an iron-dependent form of nonapoptotic cell death.. Cell.

[5] Doll S, Freitas FP, Shah R, Aldrovandi M, da Silva MC, Ingold I, Goya Grocin A, Xavier da Silva TN, Panzilius E, Scheel CH, Mourao A, Buday K, Sato M, Wanninger J, Vignane T, Mohana V, Rehberg M, Flatley A, Schepers A, Kurz A, White D, Sauer M, Sattler M, Tate EW, Schmitz W, Schulze A, O’Donnell V, Proneth B, Popowicz GM, Pratt DA (2019). FSP1 is a glutathione-independent ferroptosis suppressor.. Nature.

[6] Morales M, Xue × (2021). Targeting iron metabolism in cancer therapy.. Theranostics.

[7] Floros KV, Cai J, Jacob S, Kurupi R, Fairchild CK, Shende M, Coon CM, Powell KM, Belvin BR, Hu B, Puchalapalli M, Ramamoorthy S, Swift K, Lewis JP, Dozmorov MG, Glod J, Koblinski JE, Boikos SA, Faber AC (2021). MYCN-Amplified Neuroblastoma Is Addicted to Iron and Vulnerable to Inhibition of the System Xc-/Glutathione Axis.. Cancer research.

[8] Fry JS, Hamling JS, Lee PN (2012). Systematic review with meta-analysis of the epidemiological evidence relating FEV1 decline to lung cancer risk.. BMC Cancer.

[9] Yang WS, SriRamaratnam R, Welsch ME, Shimada K, Skouta R, Viswanathan VS, Cheah JH, Clemons PA, Shamji AF, Clish CB, Brown LM, Girotti AW, Cornish VW, Schreiber SL, Stockwell BR (2014). Regulation of ferroptotic cancer cell death by GPX4.. Cell.

[10] Combs JA, DeNicola GM (2019). The Non-Essential Amino Acid Cysteine Becomes Essential for Tumor Proliferation and Survival.. Cancers.

[11] Chaturvedi S, Hoffman RM, Bertino JR (2018). Exploiting methionine restriction for cancer treatment.. Biochem Pharmacol.

[12] Sanderson SM, Gao X, Dai Z, Locasale JW (2019). Methionine metabolism in health and cancer: a nexus of diet and precision medicine.. Nat Rev Cancer.

[13] Zhang W, Yu Y, Hertwig F, Thierry-Mieg J, Zhang W, Thierry-Mieg D, Wang J, Furlanello C, Devanarayan V, Cheng J, Deng Y, Hero B, Hong H, Jia M, Li L, Lin SM, Nikolsky Y, Oberthuer A, Qing T, Su Z, Volland R, Wang C, Wang MD, Ai J, Albanese D, Asgharzadeh S, Avigad S, Bao W, Bessarabova M, Brilliant MH (2015). Comparison of RNA-seq and microarray-based models for clinical endpoint prediction.. Genome Biol.

[14] Kocak H, Ackermann S, Hero B, Kahlert Y, Oberthuer A, Juraeva D, Roels F, Theissen J, Westermann F, Deubzer H, Ehemann V, Brors B, Odenthal M, Berthold F, Fischer M (2013). Hox-C9 activates the intrinsic pathway of apoptosis and is associated with spontaneous regression in neuroblastoma.. Cell Death Dis.

[15] Valentijn LJ, Koster J, Haneveld F, Aissa RA, van Sluis P, Broekmans ME, Molenaar JJ, van Nes J, Versteeg R (2012). Functional MYCN signature predicts outcome of neuroblastoma irrespective of MYCN amplification.. Proc Natl Acad Sci U S A.

[16] Abeles RH, Walsh CT (1973). Acetylenic enzyme inactivators. Inactivation of gamma-cystathionase, in vitro and in vivo, by propargylglycine.. J Am ChemSoc.

[17] Alvarez E, Gironès N, Davis RJ (1989). Intermolecular disulfide bonds are not required for the expression of the dimeric state and functional activity of the transferrin receptor.. EMBO J.

[18] Feng H, Schorpp K, Jin J, Yozwiak CE, Hoffstrom BG, Decker AM, Rajbhandari P, Stokes ME, Bender HG, Csuka JM, Upadhyayula PS, Canoll P, Uchida K, Soni RK, Hadian K, Stockwell BR (2020). Transferrin Receptor Is a Specific Ferroptosis Marker.. Cell Rep.

[19] Shin CS, Mishra P, Watrous JD, Carelli V, D’Aurelio M, Jain M, Chan DC (2017). The glutamate/cystine xCT antiporter antagonizes glutamine metabolism and reduces nutrient flexibility.. Nat Commun.

[20] Xia Y, Ye B, Ding J, Yu Y, Alptekin A, Thangaraju M, Prasad PD, Ding ZC, Park EJ, Choi JH, Gao B, Fiehn O, Yan C, Dong Z, Zha Y, Ding HF (2019). Metabolic Reprogramming by MYCN Confers Dependence on the Serine-Glycine-One-Carbon Biosynthetic Pathway.. Cancer Res.

[21] Gamble LD, Purgato S, Murray J, Xiao L, Yu DMT, Hanssen KM, Giorgi FM, Carter DR, Gifford AJ, Valli E, Milazzo G, Kamili A, Mayoh C, Liu B, Eden G, Sarraf S, Allan S, Di Giacomo S, Flemming CL, Russell AJ, Cheung BB, Oberthuer A, London WB, Fischer M, Trahair TN, Fletcher JI, Marshall GM, Ziegler DS, Hogarty MD, Burns MR (2019). Inhibition of polyamine synthesis and uptake reduces tumor progression and prolongs survival in mouse models of neuroblastoma.. Sci Translat Med.

[22] Bachmann AS, Geerts D (2018). Polyamine synthesis as a target of MYC oncogenes.. J Biol Chem.

[23] Zhu J, Berisa M, Schworer S, Qin W, Cross JR, Thompson CB (2019). Transsulfuration Activity Can Support Cell Growth upon Extracellular Cysteine Limitation.. Cell Metab.

[24] Al-Ani A, Toms D, Kondro D, Thundathil J, Yu Y, Ungrin M (2018). Oxygenation in cell culture: Critical parameters for reproducibility are routinely not reported.. PloS one.

[25] Anderson CP, Seeger RC, Satake N, Monforte-Munoz HL, Keshelava N, Bailey HH, Reynolds CP (2001). Buthionine sulfoximine and myeloablative concentrations of melphalan overcome resistance in a melphalan-resistant neuroblastoma cell line.. J Pediatr Hematol Oncol.

[26] Villablanca JG, Volchenboum SL, Cho H, Kang MH, Cohn SL, Anderson CP, Marachelian A, Groshen S, Tsao-Wei D, Matthay KK, Maris JM, Hasenauer CE, Czarnecki S, Lai H, Goodarzian F, Shimada H, Reynolds CP (2016). A Phase I New Approaches to Neuroblastoma Therapy Study of Buthionine Sulfoximine and Melphalan With Autologous Stem Cells for Recurrent/Refractory High-Risk Neuroblastoma.. Pediatr Blood Cancer.

[27] Higuchi T, Han Q, Sugisawa N, Yamamoto J, Yamamoto N, Hayashi K, Kimura H, Miwa S, Igarashi K, Bouvet M, Singh SR, Tsuchiya H, Hoffman RM (2021). Combination Methionine-methylation-axis Blockade: A Novel Approach to Target the Methionine Addiction of Cancer.. Cancer Genomics Proteomics.

[28] Ham J, Costa C, Sano R, Lochmann TL, Sennott EM, Patel NU, Dastur A, Gomez-Caraballo M, Krytska K, Hata AN, Floros KV, Hughes MT, Jakubik CT, Heisey DA, Ferrell JT, Bristol ML, March RJ, Yates C, Hicks MA, Nakajima W, Gowda M, Windle BE, Dozmorov MG, Garnett MJ, McDermott U, Harada H, Taylor SM, Morgan IM, Benes CH, Engelman JA (2016). Exploitation of the Apoptosis-Primed State of MYCN-Amplified Neuroblastoma to Develop a Potent and Specific Targeted Therapy Combination.. Cancer Cell.

[29] Nakagawa M, Takizawa N, Narita M, Ichisaka T, Yamanaka S (2010). Promotion of direct reprogramming by transformation-deficient Myc.. Proc Natl Acad Sci U S A.

[30] Kocak H, Ackermann S, Hero B, Kahlert Y, Oberthuer A, Juraeva D, Roels F, Theissen J, Westermann F, Deubzer H, Ehemann V, Brors B, Odenthal M, Berthold F, Fischer M (2013). Hox-C9 activates the intrinsic pathway of apoptosis and is associated with spontaneous regression in neuroblastoma.. Cell Death Dis.

[31] Kocak H, Ackermann S, Hero B, Kahlert Y, Oberthuer A, Juraeva D, Roels F, Theissen J, Westermann F, Deubzer H, Ehemann V, Brors B, Odenthal M, Berthold F, Fischer M (2014). A comprehensive assessment of RNA-seq accuracy, reproducibility and information content by the Sequencing Quality Control Consortium.. Nature Biotechnol.

[32] Mei S, Qin Q, Wu Q, Sun H, Zheng R, Zang C, Zhu M, Wu J, Shi X, Taing L, Liu T, Brown M, Meyer CA, Liu XS (2017). Cistrome Data Browser: a data portal for ChIP-Seq and chromatin accessibility data in human and mouse.. Nucleic Acids Res.

[33] Zeid R, Lawlor MA, Poon E, Reyes JM, Fulciniti M, Lopez MA, Scott TG, Nabet B, Erb MA, Winter GE, Jacobson Z, Polaski DR, Karlin KL, Hirsch RA, Munshi NP, Westbrook TF, Chesler L, Lin CY, Bradner JE (2018). Enhancer invasion shapes MYCN-dependent transcriptional amplification in neuroblastoma.. Nature Genet.

[34] Hsu CL, Chang HY, Chang JY, Hsu WM, Huang HC, Juan HF (2016). Unveiling MYCN regulatory networks in neuroblastoma via integrative analysis of heterogeneous genomics data.. Oncotarget.

[35] Robinson JT, Thorvaldsdóttir H, Winckler W, Guttman M, Lander ES, Getz G, Mesirov JP (2011). Integrative genomics viewer.. Nature Biotechnol.

[36] Guan R, Ho MC, Brenowitz M, Tyler PC, Evans GB, Almo SC, Schramm VL (2011). Entropy-driven binding of picomolar transition state analogue inhibitors to human 5’-methylthioadenosine phosphorylase.. Biochemistry.

[37] Meier M, Janosik M, Kery V, Kraus JP, Burkhard P (2001). Structure of human cystathionine beta-synthase: a unique pyridoxal 5’-phosphate-dependent heme protein.. EMBO J.

